# Lipoprotein Metabolism, Dyslipidemia, and Lipid-Lowering Therapy in Women: A Comprehensive Review

**DOI:** 10.3390/ph17070913

**Published:** 2024-07-09

**Authors:** Jakub Michal Zimodro, Magda Mucha, Heiner K. Berthold, Ioanna Gouni-Berthold

**Affiliations:** 1 1st Chair and Department of Cardiology, Medical University of Warsaw, 02-097 Warsaw, Poland; 2 Faculty of Medicine, Medical University of Bialystok, 15-089 Bialystok, Poland; 3 Department of Internal Medicine and Geriatrics, Bethel Clinic (EvKB), 33611 Bielefeld, Germany; 4 Center for Endocrinology, Diabetes and Preventive Medicine, Faculty of Medicine, University Hospital Cologne, University of Cologne, Kerpener Str. 62, 50937 Cologne, Germany

**Keywords:** atherosclerotic cardiovascular disease, cholesterol, dyslipidemia, lipid-lowering therapy, women, sex differences

## Abstract

Lipid-lowering therapy (LLT) is a cornerstone of atherosclerotic cardiovascular disease prevention. Although LLT might lead to different reductions in low-density lipoprotein cholesterol (LDL-C) levels in women and men, LLT diminishes cardiovascular risk equally effectively in both sexes. Despite similar LLT efficacy, the use of high-intensity statins, ezetimibe, and proprotein convertase subtilisin/kexin type 9 inhibitors is lower in women compared to men. Women achieve the guideline-recommended LDL-C levels less often than men. Greater cholesterol burden is particularly prominent in women with familial hypercholesterolemia. In clinical practice, women and men with dyslipidemia present with different cardiovascular risk profiles and disease manifestations. The concentrations of LDL-C, lipoprotein(a), and other blood lipids differ between women and men over a lifetime. Dissimilar levels of LLT target molecules partially result from sex-specific hormonal and genetic determinants of lipoprotein metabolism. Hence, to evaluate a potential need for sex-specific LLT, this comprehensive review (i) describes the impact of sex on lipoprotein metabolism and lipid profile, (ii) highlights sex differences in cardiovascular risk among patients with dyslipidemia, (iii) presents recent, up-to-date clinical trial and real-world data on LLT efficacy and safety in women, and (iv) discusses the diverse medical needs of women and men with dyslipidemia and increased cardiovascular risk.

## 1. Introduction

Atherosclerotic cardiovascular disease (ASCVD) remains the leading cause of death in women and men worldwide [1]. Given the fundamental role of blood lipoproteins in atherosclerotic plaque formation, hypercholesterolemia and hypertriglyceridemia substantially contribute to ASCVD development and progression [2]. Therefore, lipid-lowering therapy (LLT) is a mainstay of ASCVD prevention and treatment [3]. In women and men with increased low-density lipoprotein cholesterol (LDL-C) levels, according to the patient’s global cardiovascular risk, currently recommended treatment regimens comprise [high-intensity] statins, ezetimibe, bempedoic acid, and proprotein convertase subtilisin/kexin type 9 (PCSK9) inhibitors [4,5]. In addition, novel therapeutic targets such as lipoprotein(a) [Lp(a)] [6], and novel drug classes, such as antisense oligonucleotides and small interfering ribonucleic acids, are intensively studied [7,8].

Real-world data demonstrate that women less often receive the guideline-recommended LLT [9,10,11,12] and thus do not benefit from ASCVD prevention strategies equally to men. Sex differences exist in lipoprotein metabolism, most likely due to sex-specific hormonal and genetic determinants [13,14]. The potential mechanisms leading to the different female and male lipid profiles [15], especially among patients with familial hypercholesterolemia (FH) [16], are still a matter of discussion. Furthermore, women have additional risk factors related to reproductive health and psychosocial status that are not present in men [17,18]. Among patients with dyslipidemia, sex disparities are also observed in LDL-C goal attainment [19,20,21], LLT-related side effects [22], adherence to LLT [23,24], perception of dyslipidemia and cardiovascular risk, as well as patient–physician relationship [25]. Hence, sex can affect the diagnostic process, treatment course, and clinical outcome. Importantly, differences may be driven not only by biological but also psychosocial characteristics. Therefore, along with sex, the significance of gender cannot be neglected [17,18].

The purpose of this work, based on a selective PubMed search for up-to-date evidence, is to review (i) the impact of sex on lipoprotein metabolism, (ii) sex differences in lipid profiles, (iii) the cardiovascular risk and disease manifestation in women with dyslipidemia in comparison to men, (iv) the LLT strategies, along with their efficacy and safety in women, considering data from clinical trials and real-world studies, and (v) a potential need for sex-specific recommendations regarding LLT to overcome challenges faced in clinical practice due to the diverse medical needs of women and men with dyslipidemia and increased cardiovascular risk.

## 2. Sex Differences in Lipoprotein Metabolism

### 2.1. Role of Sex Hormones

**Figure 1:**
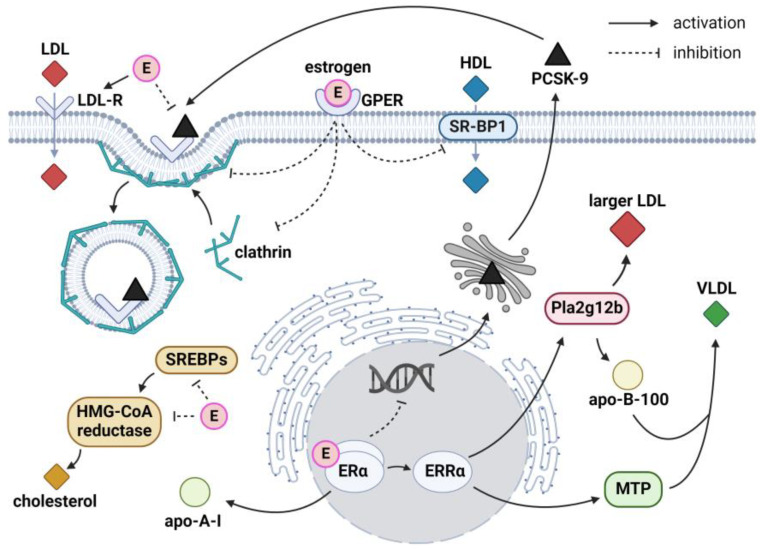
Impact of estrogens on hepatic lipoprotein metabolism. The arrows indicate the activation or inhibition of selected molecular processes involved in hepatic lipoprotein metabolism. Apo—apolipoprotein; E—estrogens, ERα—estrogen receptor alpha; ERRα—estrogen-related receptor alpha; GPER—G protein-coupled estrogen receptor; HDL—high-density lipoprotein; HMG-CoA—3-hydroxy-3-methyl-glutaryl-coenzyme A; LDL—low-density lipoprotein; LDL-R—low-density lipoprotein receptor; MTP—microsomal triglyceride transfer protein; PCSK9—proprotein convertase subtilisin/kexin type 9; Pla2g12b—phospholipase A2 group XII B; SREBPs—sterol regulatory element-binding proteins; VLDL—very-low-density lipoprotein. Created with BioRender.com.

ASCVD develops 7–10 years later in women compared to men [26]. Cardiovascular risk in women increases following menopause, accompanied by a decline in the production and concentration of estrogens, the primary female sex hormones. Estrogens exert a cardioprotective effect and hence contribute to sex differences in cardiovascular risk [27]. Among their multi-faceted actions, estrogens seem to affect the lipid profile and its atherogenicity [13,14]. The following sections describe the basic effects of sex hormones on molecular mechanisms studied in humans, as well as tissue and animal models. The impact of estrogens on hepatic lipoprotein metabolism is summarized in Figure 1.

#### 2.1.1. Estrogens and Very-Low-Density Lipoproteins

Estrogens affect triglyceride-rich very-low-density lipoprotein (VLDL-TG) metabolism. First, estrogen-related receptor alpha (ERRα), a downstream target of estrogen receptor alpha (ERα), upregulates apolipoprotein (apo) B-100 and microsomal triglyceride transfer protein (MTP) expression. Consequently, ERRα increases VLDL-TG production and secretion in the liver [28]. Correspondingly, in the presence of cholesteryl ester transfer protein (CETP), estrogens enhance small heterodimer partner and protein disulfide isomerase expression, leading to increased VLDL-TG production. Of note, the effect of estrogens is mediated not only via ERα but also via the G protein-coupled estrogen receptor (GPER) [29].

Women exhibit a higher VLDL-TG secretion rate, which, along with a lower VLDL-apoB-100 secretion rate, suggests that women secrete fewer but TG-richer VLDLs, which might be more prone to hydrolysis by lipoprotein lipase due to the larger size [30,31]. In fact, a higher VLDL-TG secretion rate in women is counteracted by an accelerated clearance rate [32]. Considering the relationship with sex hormones, in postmenopausal women on hormone replacement therapy (HRT), the administration of oral estrogen increases VLDL-TG production, whereas oral progestin and transdermal estrogen stimulate VLDL-TG clearance [14,33]. However, no changes in VLDL-TG and VLDL-apoB-100 kinetics occur during the menstrual cycle [34].

#### 2.1.2. Estrogens and Low-Density Lipoproteins

Estrogens play a key role in LDL-C metabolism. Estrogens increase LDL receptor (LDL-R) expression and activity on the liver surface via ERα, leading to increased LDL-C uptake and subsequent decline in circulating LDL-C levels [35,36,37].

The effect on the LDL-R is also mediated by the impact of estrogens on the expression and function of proprotein convertase subtilisin/kexin type 9 (PCSK9). Following binding to the LDL-R extracellular domain, PCSK9 triggers LDL-R endocytosis and lysosomal degradation through the clathrin trafficking pathway [38,39]. Overall, higher circulating PCSK9 levels are found in women compared to men, as well as in postmenopausal compared to premenopausal women [40]. In addition, circulating PCSK9 levels differ between menstrual cycle phases, with an inverse correlation between PCSK9 and estrogen levels [41,42]. However, this association is observed only for endogenous estrogens, as HRT has no effect on PCSK9 levels in postmenopausal women [40,43]. Furthermore, an in vitro study revealed that the phytoestrogen resveratrol suppresses PCSK9 proximal promoter via ERα, resulting in lower PCSK9 production [44]. Conversely, other studies suggested that the impact of estrogens on PCSK9 production through genetic signaling is nonsignificant, contrary to the impact on PCSK9 clearance [40,45,46].

Several studies examined functional modifications of PCSK9 on cell lines. First, estrogens inhibit the phosphorylation of secreted PCSK9, impeding its interaction with LDL-R [45]. Furthermore, estrogens block PCSK9 internalization and alter clathrin distribution, resulting in the inhibition of LDL-R endocytosis. Interestingly, estrogens additionally enhance LDL-C uptake in the presence of extracellular PCSK9. Of note, both effects are mediated via GPER [47,48]. Conversely, a complex relationship occurs between estrogens and PCSK9 distribution, as PCSK9-deficient male mice exhibit higher LDL-R levels on the liver surface compared to female mice [46]. Correspondingly, in hepatic cells treated with hyperlipidemic serum of postmenopausal women, estrogens reduce LDL-C uptake, and thus, diminish hepatic lipid accumulation. This effect is mediated by sterol regulatory element-binding protein-1 (SREBP-1), the expression of which is promoted by ERα [49].

Moreover, estrogens influence lipoprotein size as ERRα stimulates phospholipase A2 group XII B (Pla2g12b) expression. Mutation in the Pla2g12b gene reduces the levels of apoB and large lipoproteins but significantly increases the concentration of small LDL-like particles [13,50]. Correspondingly, certain variations in the ERα gene are associated with higher levels of small LDL particles of greater atherogenicity [51,52], accompanied by increased risk of myocardial infarction (MI) [53] and aortic valve stenosis [54].

The anti-atherogenic effects of estrogens are also exerted independently of hepatic LDL-C metabolism. Estrogens reduce LDL-C uptake in foam cells through a pathway involving ERα and SREBP-1 signaling and hence inhibit foam cell formation. Importantly, estrogens increase the sensitivity to statin treatment as the combination of estrogen with rosuvastatin reduces lipid accumulation in foam cells compared to statin monotherapy, as well as counteracts atherosclerotic plaque progression [49]. Estrogen-induced reduction in total cholesterol (TC) levels and inhibition of atherosclerotic plaque formation is mediated by ERα [55]. Considering other receptors, scavenger receptor class B type 1 (SR-B1) enhances LDL transcytosis in coronary endothelial cells, as investigated using a novel assay [56]. This effect is opposed by estrogens, which inhibit SR-B1 expression via GPER [57]. Noteworthy, the anti-atherogenic effect of GPER was proposed as ligand-independent, indicating its crucial role in maintaining cardiovascular health [58]. Finally, although no differences in LDL oxidation occur during the menstrual cycle [59], a protective effect of estrogens against LDL oxidation was proposed [60].

#### 2.1.3. Estrogens and High-Density Lipoproteins

Estrogens are involved in high-density lipoprotein cholesterol (HDL-C) metabolism and reverse cholesterol transport. Estrogens reduce concentrations of hepatic SR-B1, impeding HDL-C uptake and subsequently increasing circulating HDL-C levels [61]. The effect of estrogens on hepatic SR-B1 expression is likely post-transcriptional, as authors have reported no [62] or a lower decrease [57] in the concentration of messenger ribonucleic acid for SR-B1 compared to SR-B1 protein levels. Of note, the decline in SR-B1 levels was linked to an estrogen-associated increase in LDL-R activity [62].

Considering further aspects of reverse cholesterol transport, administration of oral estrogen and progestin to postmenopausal women increases the expression of adenosine triphosphate-binding cassette transporter A1 (ABCA1), a protein involved in HDL-C efflux, in leukocytes [63]. Similarly, estrogens enhance cholesterol efflux from vascular smooth muscle cells to apoA-I and HDL through the upregulation of ABCA1 and ABCG1 [64]. However, ABCA1-specific cholesterol efflux capacity increases in early menopause [65]. Furthermore, premenopausal women have higher CETP levels, which positively correlate with estrogen levels [66]. Interestingly, CETP is necessary for estrogen to enhance HDL-C efflux [67]. Moreover, the activity of lecithin:cholesterol acyltransferase (LCAT), a protein participating in HDL-C efflux, increases in postmenopausal women on HRT [68]. Estrogens also promote the synthesis of apoA-I but not apoA-II, with the former being a cofactor for LCAT and the latter inhibiting lipoprotein lipase activity [69,70]. In addition, estrogens inhibit hepatic lipase activity, possibly contributing to higher levels of HDL2, particles of lower density than HDL3, in women [71]. Moreover, HDL-associated estrogen fatty acyl esters enhance cholesterol efflux from macrophages via ERs and SR-B1 [72]. However, the latter results could not be confirmed in a bigger cohort of pre- and postmenopausal women and men, in which estrogens did not affect the activity of CETP, LCAT, or phospholipid transfer protein [73].

#### 2.1.4. Estrogens and Cholesterol Metabolism

Estrogens are involved in cholesterol synthesis and absorption [14]. SREBPs promote a cholesterol synthesis pathway involving 3-hydroxy-3-methyl-glutaryl-coenzyme A (HMG-CoA) reductase, the main target of statins. As demonstrated in animal models, women have lower activity and concentrations of both SREBPs and HMG-CoA reductase than men, which is associated with the presence of estrogens [14,74]. Conversely, estrogens upregulate the expression of Niemann-Pick C1-like protein 1, a target of ezetimibe, promoting increased cholesterol absorption in the intestine [75]. In addition, ERRα enhances apoA-IV expression and thus participates in the regulation of intestinal lipid absorption [76].

### 2.2. Role of Genetics

Along with sex hormones, lipoprotein metabolism is altered by an individual’s genetic component. In hyperlipidemic mice, both gonadal and chromosomal sex affect the hepatic transcriptome and the expression of genes involved in fatty acid metabolism. Interestingly, following statin treatment, a compensatory overexpression of genes involved in cholesterol synthesis is observed only in subjects with the male XY genotype [77]. A study in obese humans identified sex differences in the hepatic methylome and gene expression on autosomes and the X-chromosome. For instance, higher HDL-C levels in women correlate with greater expression of the lysine demethylase 6A gene, the silencing of which reduces apoA-I expression and HDL-C levels [78]. In addition, certain sex-specific single-nucleotide polymorphisms in CETP and apoA-V genes are related to dyslipidemia [79].

Furthermore, the presence of two X-chromosomes correlates with elevated HDL-C levels and HDL-apoA-I exchange activity. However, higher apoA-I, apoA-IV, and apoE levels were identified in subjects with the XY genotype. Moreover, the XY genotype is associated with higher TG levels compared to the XX genotype in mice on a cholesterol-enriched diet [80]. Similarly, women with one X-chromosome exhibit higher LDL-C and triglyceride (TG) levels, as well as smaller LDL and HDL particle sizes compared to women with two X-chromosomes [81]. These results would indicate a favorable impact of female genotype on lipoprotein levels. Conversely, another study reported an association between the XX genotype and greater apoB-100 synthesis, higher TC and TG levels, as well as increased expression of genes involved in intestinal lipid absorption [82].

### 2.3. Key Messages

Altogether, lipoprotein metabolism is affected by sex hormones via multiple molecular pathways. Estrogens appear to (i) counteract hepatic accumulation of fatty acids and lipids by promoting VLDL-TG production and secretion, thereby protecting against hepatic steatosis, (ii) reduce LDL-C levels by stimulating LDL-R activity, partially due to the interaction with PCSK9, (iii) promote lipoprotein size expansion, (iv) increase HDL-C levels by impeding SR-B1 activity, (v) enhance HDL-C overall efflux capacity, and (vi) modulate cholesterol synthesis and absorption of dietary lipids. Moreover, lipoprotein metabolism is influenced by sex-specific genetic variations and sex chromosomes.

## 3. Sex Differences in Lipid Profiles

### 3.1. Lipid Profile throughout the Lifetime

**Figure 2:**
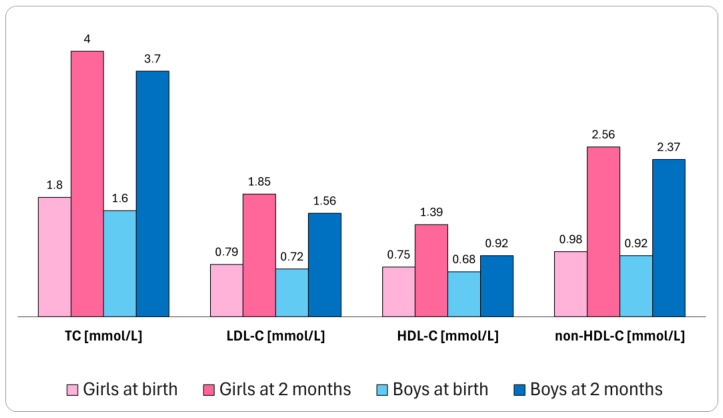
Sex differences in lipid profiles early in life. The median concentrations presented were derived from [84]. HDL-C—high-density lipoprotein cholesterol; LDL-C—low-density lipoprotein cholesterol; TC—total cholesterol.

Lipid profiles vary with age and differ between women and men, which has recently been the subject of extensive reviews [15,17,83]. In the general population, higher TC (median: 1.8 vs. 1.6 mmol/L, *p* < 0.001), LDL-C (0.79 vs. 0.72 mmol/L, *p* < 0.001), HDL-C (0.75 vs. 0.68 mmol/L, *p* < 0.001), non-HDL-C (0.98 vs. 0.92 mmol/L, *p* < 0.05), and apoB levels (31 vs. 29 mg/dL, *p* < 0.001) were found in girls compared to boys in cord blood at birth, with corresponding results observed when measured in venous blood. Similar trends in TC (median: 4.0 vs. 3.7 mmol/L, for women and men, respectively, *p* < 0.001), LDL-C (1.85 vs. 1.56 mmol/L, *p* < 0.05), HDL-C (1.39 vs. 1.23 mmol/L, *p* < 0.001), non-HDL-C (2.56 vs. 2.37 mmol/L, *p* < 0.05), and apoB levels (76 vs. 71 mg/dL, *p* < 0.05) were also reported at 2 months but not at 14–16 months of age, as presented in Figure 2 [84].

During infancy, higher TC and LDL-C levels can be found in girls up to 6 months of age. Less pronounced and inconsistent sex differences might also occur in HDL-C and TG levels [85]. In childhood and early adolescence, girls exhibit higher TC, LDL-C, non-HDL-C, and apoB levels [15]. Starting at the age of around 20 years, men are believed to exhibit more atherogenic lipid profiles, with elevated LDL-C and TG levels [15]. However, several studies reported higher levels of Lp(a), a molecule of six-fold greater atherogenicity compared to LDL on a per-particle basis, in women [86], especially in those diagnosed with ASCVD [87,88].

Among newborns of mothers with hypercholesterolemia, girls exhibit higher TC, LDL-C, HDL-C, apoB, and apoA-I levels in cord blood compared to boys [89]. Considering hereditary lipid disorders in children with FH, increased TC (mean difference [MD]: 0.48 mmol/L, *p* < 0.001), LDL-C (0.39 mmol/L, *p* < 0.001), and non-HDL-C (0.42 mmol/L, *p* < 0.001) levels were demonstrated in girls. In the same group, higher HDL-C levels were found in girls aged <5 years (mean 1.25 vs. 1.10 mmol/L, *p* < 0.05) or 15–19 years (1.34 vs. 1.09 mmol/L, *p* < 0.001) compared to boys of the same age [90]. Importantly, contrary to the general population, among patients with heterozygous familial hypercholesterolemia (HeFH), higher TC (median: 8.2 vs. 7.6 mmol/L, *p* < 0.001 [91]) and LDL-C levels (6.2 vs. 6.0 mmol/L, *p* = 0.005 [92]) were described in women. However, similar untreated TC (mean: 645 vs. 650 mg/dL ≈ 16.68 vs. 16.81 mmol/L, for women and men, respectively, *p* = 0.8) and LDL-C levels (579 vs. 596 mg/dL ≈ 14.97 vs. 15.41 mmol/L, *p* = 0.34) were found in women and men with homozygous familial hypercholesterolemia (HoFH) at a median age of 13 years [93].

### 3.2. Impact of Hormonal Status in Women

#### 3.2.1. Menstrual Cycle

Small variations in the lipid profile have been reported during the menstrual cycle [15,83], with a non-linear association between the menstrual cycle phase and TC, LDL-C, and HDL-C levels [83,94]. Specifically, the highest TC and LDL-C levels were identified in the follicular phase, which is characterized by low estrogen levels. The median TC and LDL-C levels decrease on average by 3% and 4.9% (*p* < 0.0001), respectively, in the luteal phase. Furthermore, the highest HDL-C levels occur during ovulation with an increase of 2% compared to other phases [95]. Considering other lipid parameters, although lower TG and very low-density lipoprotein cholesterol (VLDL-C) levels were described in the luteal phase, the decrease was not statistically significant [96,97]. Importantly, in a study of 259 healthy women, when measured in the late luteal phase, 10.5% of the participants had LDL-C levels above the desirable range defined as <130 mg/dL, compared to 17.8% when measured in the follicular phase, showing important clinical implications, especially when women of reproductive age are included in clinical trials [95].

#### 3.2.2. Pregnancy

Pregnancy is associated with elevated lipoprotein levels as lipids are crucial for fetal development [15,83]. Due to altered hepatic and adipose metabolism, TC, LDL-C, HDL-C, and TG levels continue to increase from the first trimester and peak at the end of the second trimester. Therefore, dyslipidemia is likely most pronounced during the second and third trimesters. Interestingly, a similar increase in lipoprotein levels is observed in pregnant women with and without a history of dyslipidemia [98,99], whereas higher absolute LDL-C levels occur in women with HeFH, reaching values >8 mmol/L in the 30th gestational week [16]. In a study of 222 pregnant women, 60% of the participants reached LDL-C levels above the value recommended for non-pregnant patients [100]. In addition, LDL size decreases during pregnancy, leading to increased LDL oxidation and higher atherogenicity [101]. However, HDL-C and apoA-I levels also gradually increase, with a peak in the second trimester. Nonetheless, the greatest increase during pregnancy affects TG levels, reaching two- to four-fold higher values compared to pre-pregnancy. Postnatally, lipoprotein levels normalize within 6 weeks to 6 months [15,99]. Interestingly, these changes might be influenced by breastfeeding [15,102]. Specifically, women who breastfeed have lower LDL-C levels and experience smaller declines in HDL-C levels [103], preserving higher HDL-C values during 6 months of lactation [104].

#### 3.2.3. Menopause

Due to a decline in estrogen production, the lipid profile becomes more atherogenic following menopause [15,83]. Elevated TC, LDL-C, and apoB levels were described within a year interval from the final menstrual period. Importantly, the increase was independent of age [105]. Furthermore, a meta-analysis revealed increased TC (MD: 0.58 mmol/L), LDL-C (0.45 mmol/L), and TG (0.27 mmol/L) levels in postmenopausal women. Although the change was partially attributable to older age [106], when adjusted for age and body mass index, postmenopausal women had higher TC levels compared to premenopausal women (mean: 5.2 vs. 4.94 mmol/L, *p* < 0.045) [107]. In addition, higher LDL-C (mean: 2.90 vs. 2.73 mmol/L, *p* = 0.013) and lower HDL-C levels (1.47 vs. 1.55 mmol/L, *p* = 0.013) were found in women who experienced menopause >6 years prior compared to those who had menopause <2 years prior [108]. On the contrary, a meta-analysis found no differences in HDL-C levels between pre- and postmenopausal women [106]. Intriguingly though, inconsistent study results suggest that high HDL-C levels must not necessarily be cardioprotective in middle-aged women [109]. Finally, women experience an increase in Lp(a) levels around the age of 50 years, with even 22% higher Lp(a) levels following menopause [110], which substantially contributes to a greater atherogenicity of the lipid profile.

Considering the loss of ovarian function, women with premature ovarian insufficiency (POI), defined as menopause before the age of 40 years, are at increased cardiovascular risk [111]. An association between POI and higher TG and marginally lower HDL-C levels was found [112]. A meta-analysis confirmed these results and reported higher TC, LDL-C, and TG levels in patients with POI compared to healthy controls but did not identify any differences in HDL-C levels [113].

#### 3.2.4. Female-Specific Comorbidities

Women with polycystic ovary syndrome (PCOS) have an increased risk of developing metabolic syndrome and dyslipidemia. PCOS is characterized by excessive androgen production [15,83]. In women, dehydroepiandrosterone negatively correlates with TC levels, whereas androstenedione is associated with lower TG and apoA levels [114]. A recent meta-analysis of 23 cohort studies found higher TC and lower HDL-C levels in women with PCOS compared to healthy controls [115]. Furthermore, another meta-analysis found no differences in HDL-C but described higher TG levels in obese women with PCOS compared to obese women without PCOS [116]. Regarding another female-specific comorbidity, endometriosis is associated with an unfavorable lipid profile. Higher levels of all lipoproteins were found in women with endometriosis compared to healthy controls, with the most pronounced increase in LDL-C levels (MD: 37%, *p* < 0.0001) [117].

### 3.3. Impact of Exogenous Estrogens

#### 3.3.1. Contraception

Contraceptives containing sex hormones affect the lipid profile [15,83]. The impact of contraception differs regarding the content of estrogen and progestin. The estrogen component is believed to reduce LDL-C levels, as well as to increase HDL-C and TG levels, whereas the progestin component exhibits the opposite effect, depending on its androgenicity [15,118]. Traditionally used ethinyl estradiol has a greater impact on hepatic lipoprotein metabolism compared to the novel estetrol [119]. A combination of ethinyl estradiol and desogestrel, a third-generation progestin, reduces LDL-C and increases HDL-C levels [120]. On the contrary, a modern combination of estetrol and drospirenone, a fourth-generation progestin, has no significant effect on TC, LDL-C, and HDL-C levels, and causes a lower increase in TG levels compared to a combination of ethinyl estradiol and drospirenone (MD: 24% vs. 65.5% [121]; and 10% vs. 61.2% [122]). Interestingly, a 29.7% decline in TG levels was observed with a combination of estetrol and levonorgestrel, a second-generation progestin [122], compared to a 28% increase in TG levels with a combination of ethinyl estradiol and levonorgestrel [121]. Regarding the form of combined contraception, no differences were found between oral contraceptives and vaginal rings [123] or dermal patches [118,124].

Considering the progestin component of contraception, more androgenic progestins increase LDL-C and decrease HDL-C levels, contrary to the more favorable impact of novel, less androgenic progestins [118,119]. Progesterone-only pills with drospirenone or desogestrel are associated with only statistically nonsignificant declines in TC, LDL-C, HDL-C, and TG levels, whereas a significantly stronger reduction in TG levels was reported for drospirenone (MD: 0.226 vs. 0.111 mmol/L, *p* = 0.0351) [119]. In addition, a decline in Lp(a) levels was described in women on desogestrel-containing contraceptives, with lower Lp(a) levels found in oral contraception users compared to non-users (median: 4.5 vs. 3.8 mg/dL, *p* = 0.008) [125]. In another study though, the overall effect of a desogestrel-only pill was comparable to levonorgestrel and was described as negligible [126]. Considering other administration routes, levonorgestrel-releasing intrauterine device reduces TC (mean: 4.94 vs. 4.66 mmol/L, *p* = 0.037) and TG levels (0.93 vs. 0.77 mmol/L, *p* = 0.021) within 12 months of use. Although a decline in HDL-C levels can be observed at 6 months following insertion (1.38 vs. 1.29 mmol/L, *p* = 0.012), values at 12 months are similar to those measured before insertion. Finally, a non-hormonal intrauterine device containing copper shows no effect on the lipid profile throughout 18 months of use [127].

#### 3.3.2. Hormone Replacement Therapy

Regarding postmenopausal women, HRT reduces TC, LDL-C [127], Lp(a) [110,128,129,130], and apoB levels [129]. A favorable impact of exogenous estrogens on the lipid profile was found among women with POI, with those on HRT having similar TC, VLDL-C, LDL-C, and TG levels compared to healthy controls. In addition, the former group had higher HDL-C levels than the latter (mean: 1.45 vs. 1.34 mmol/L, *p* = 0.03) [131]. Interestingly, although oral HRT significantly decreases LDL-C levels, it also leads to higher TG levels compared to transdermal HRT, which has a more neutral effect on TG levels [128,132]. However, oral HRT is more effective in reducing Lp(a) levels (MD: 20.35%; *p* < 0.0001), whereas no differences depending on estrogen dose or progestin addition were identified [130]. Conversely, the effect of HRT on TG levels is influenced by estrogen dose, as a meta-analysis found a correlation between low-dose estrogen and lower TG levels compared to standard-dose estrogen. Considering HRT formulation, a combination with progestin is associated with higher TC and LDL-C levels compared to estrogen alone [128].

### 3.4. Key Messages

Altogether, sex differences in the lipid profile can be found throughout life. Girls exhibit elevated TC and LDL-C levels in childhood compared to boys. Although the opposite is likely to be observed during adolescence in the general population, among patients with HeFH, higher TC and LDL-C levels are found in women. Moreover, variations in female lipid profiles occur due to changes in hormonal status. During the menstrual cycle, TC and LDL-C levels increase in the follicular phase, whereas HDL-C levels peak during ovulation. Furthermore, elevated levels of TG, followed by TC, LDL-C, and HDL-C can be found during pregnancy. After menopause, the lipid profile becomes more atherogenic due to increased LDL-C, Lp(a), and TG levels. Unfavorable changes in lipid profile are also driven by female comorbidities such as POI, PCOS, and endometriosis.

Considering exogenous sex hormones, combined hormonal contraception might favorably affect LDL-C and HDL-C levels but also increase TG levels. A combination of novel estrogens and progestins of low androgenicity, as well as progesterone-only pills and non-hormonal contraception, seems to have a more neutral effect on lipid profiles. Nevertheless, available data are inconsistent, and further studies are required to assess the impact of contraception on the lipid profile. Correspondingly, HRT reduces TC, LDL-C, and Lp(a) levels but also increases TG levels in postmenopausal women. However, the unfavorable effect on TG levels is less pronounced with low-dose estrogens and transdermal HRT.

## 4. Women with Dyslipidemia in Clinical Practice

### 4.1. Patient Characteristics

#### 4.1.1. Lipid Profile and Cardiovascular Risk in Women

Sex differences observed in molecular mechanisms and lipid profiles translate into differences in cardiovascular risk. First, lipoprotein levels during infancy and childhood correlate with adult concentrations. Therefore, it was hypothesized that higher lipoprotein levels observed in girls increase the lifetime cardiovascular risk in women [84]. Indeed, among patients with HeFH, elevated LDL-C levels observed in women throughout life contribute to a greater LDL-C burden, which is the product of LDL-C concentration and exposure. Specifically, in a study involving 438 subjects with HeFH, women had higher LDL-C burden at the ages of 19 (111.7 vs. 100.7 mmol/L-years, *p* < 0.001) and 30 years (175.1 vs. 156.7 mmol/L-years, *p* < 0.05) compared to men. In addition, the threshold of 125 mmol/L-year, which was associated with an increased risk of MI, was reached by all women at the age of 33 years and by all men by the age of 40 years. Correspondingly, at the age of 36 years, 43.8% of women reached the threshold of 220 mmol/L-years, an average LDL-C burden at the time of MI, compared to 6.2% of men [91].

Considering other blood lipids, due to an increase in Lp(a) levels during menopausal transition, elevated Lp(a) levels are more prevalent in women than men after the age of 50 years. Therefore, Lp(a) may account for increased cardiovascular risk in postmenopausal women. In addition, a stronger association between Lp(a) and ASCVD was found in women with HeFH, compared to men with HeFH regardless of age [133]. Interestingly, among patients with type 2 diabetes mellitus (DM), cardiovascular risk declines in women following adjustment for Lp(a) levels [134]. Correspondingly, an independent association between Lp(a) levels, mortality, and non-fatal MI was found in women with type 2 DM but not in men with type 2 DM or patients without type 2 DM [135]. Hence, an increased Lp(a) level is an essential cardiovascular risk factor in women, especially in combination with elevated TC [136] or LDL-C levels [88]. Moreover, although hypertriglyceridemia was linked to increased cardiovascular risk in both sexes, non-fasting TG levels have a stronger prognostic value for predicting MI, coronary artery disease (CAD), total mortality [137], and peripheral artery disease (PAD) [138] in women compared to men. Hence, it was suggested that elevated TG levels are a more potent cardiovascular risk factor in women [17].

#### 4.1.2. Cardiovascular Risk Factors in Women

**Figure 3:**
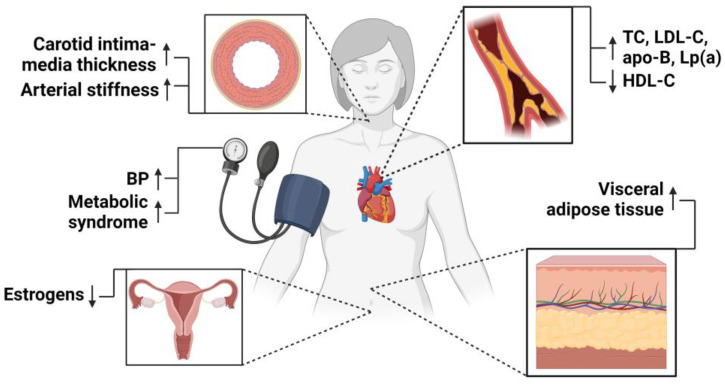
Cardiovascular risk factors in postmenopausal women. The arrows indicate an increase or a decline in the prevalence of cardiovascular risk factors after menopausal transition. Apo—apolipoprotein; BP—blood pressure; LDL-C—low-density lipoprotein cholesterol; Lp(a)—lipoprotein(a); TC—total cholesterol. Created with BioRender.com.

Regarding the patient’s global cardiovascular risk, women treated for ASCVD are likely to be older and have more cardiovascular risk factors than men due to the later onset of ASCVD [17]. The prevalence of dyslipidemia, glucose intolerance, visceral adiposity, and arterial hypertension increases following menopause, leading to endothelial dysfunction and inflammation, which in turn predispose to ASCVD [27]. A decline in estrogen levels is also associated with the up-regulation of the renin–angiotensin–aldosterone system and the sympathetic nervous system, as well as with reduced nitric oxide-dependent vasodilation, which alters vascular function [139]. The cardiovascular risk profile of postmenopausal women is presented in Figure 3. Women are also more often affected by systemic autoimmune disorders, which promote ASCVD development and progression [140]. Interestingly, a greater impact of established cardiovascular risk factors, such as arterial hypertension, DM, and smoking, on global cardiovascular risk was reported in women [18]. For instance, a stronger association between first MI and arterial hypertension (odds ratio (OR): 2.87 vs. 2.19, *p* < 0.001 for all), DM (3.59 vs. 1.76), and current smoking (3.28 vs. 3.25) was found in women compared to men [141].

Furthermore, sex-specific conditions accounting for increased cardiovascular risk were described and comprise obstetric and gynecological disorders, such as gestational DM or hypertension, pre-eclampsia, placental abruption, preterm delivery, stillbirth, miscarriage, premature menopause, or PCOS [18,142]. Furthermore, the use of combined oral contraception was linked to negative changes in cardiovascular risk factors [143] and increased incidence of stroke [144] and MI, especially in women with arterial hypertension or dyslipidemia [142]. Correspondingly, an association between HRT and increased cardiovascular risk was found in postmenopausal women, especially with HRT initiated years after menopause [18]. On the contrary, a favorable impact of HRT on cardiovascular risk and mortality was suggested provided HRT is started around menopausal transition [145]. Finally, increased cardiovascular risk and ASCVD-related mortality rates were found in women with breast cancer varying based on cancer treatment received [146].

Along with sex, cardiovascular risk is influenced by sociological, psychological, and cultural features reflected by gender. Due to low socioeconomic status, poor education, and limited access to healthcare, women are less likely to seek medical attention and are less aware of their own cardiovascular risk. Furthermore, women suffer from greater mental stress, depression, and anxiety, which predispose to ASCVD, and affect disease manifestation and clinical outcome [147]. A stronger association between depression and first MI was found in women than in men (OR: 3.09 vs. 1.77; *p* < 0.001), highlighting the importance of psychosocial cardiovascular risk factors in women [141].

#### 4.1.3. Atherosclerotic Cardiovascular Disease in Women

Sex differences occur in ASCVD manifestations. Women tend to have smaller atherosclerotic plaques with a lower volume of lipid-rich necrotic core and calcification in the carotid arteries [148]. Those in the coronary arteries have additionally lower volumes of fibrous tissue and fibro-fatty tissue, along with greater total plaque burden [149]. Overall, women have less vulnerable atherosclerotic plaques, and contrary to men, experience acute plaque rupture less frequently than plaque erosion [150]. Moreover, women are less likely to exhibit diffuse CAD or severe ischemia but present with more frequent angina and microvascular disease as the underlying cause [151].

Considering clinical presentation, women with acute coronary syndrome tend to show prodromal and atypical symptoms, which might be underestimated by physicians, leading to delays in treatment [152]. Specifically, a longer time from the onset of symptoms to alarming emergency medical services and to first medical contact, along with lower rates of percutaneous coronary interventions and longer in-hospital delays, followed by worse clinical outcomes, were reported in women among patients with acute coronary syndrome [153]. Similarly, women with PAD less often experience intermittent claudication and present with atypical symptoms, resulting in lower sensitivity of ankle–brachial index measurement, a more challenging diagnostic process, and delayed diagnosis. Consequently, women with PAD present with multivessel disease and chronic limb ischemia more frequently and experience a higher rate of complications after surgery, either endovascular or open, than men [154].

### 4.2. Lipid-Lowering Therapy

#### 4.2.1. Effectiveness of Lipid-Lowering Agents in Women

**Figure 4:**
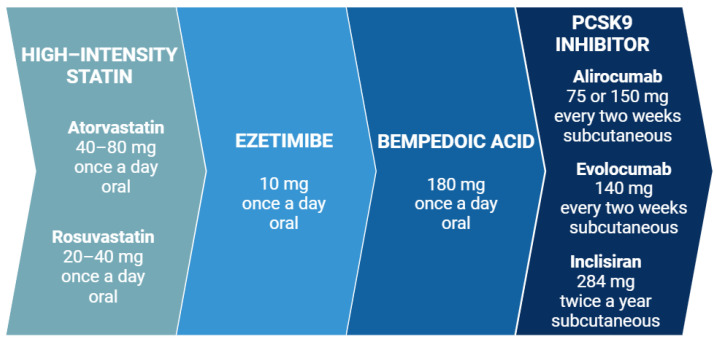
A course of lipid-lowering therapy. If a patient does not achieve the guideline-recommended low-density lipoprotein cholesterol levels, treatment intensification is based on adding a new drug in the presented order. PCSK9—proprotein convertase subtilisin/kexin type 9. Created with BioRender.com.

Reaching the guideline-recommended LDL-C levels is essential for ASCVD prevention and treatment. A strategy for LLT intensification is presented in Figure 4. Currently, first-line LLT includes a statin in a maximally tolerated dose [3]. Although a study on 337 patients found lower LDL-C reduction in women after adjustment for dose and statin power (22.7 vs. 28.5%, *p* < 0.001) [155], a meta-analysis including 174,000 patients showed that statins are similarly efficient in reducing LDL-C levels and the risk of major adverse cardiovascular events (MACE) at 1-year follow-up in both women and men [156]. In addition, a higher increase in HDL-C levels was described in women compared to men on statin treatment (MD: 5.64%, *p* = 0.001) [157].

Considering the next step of LLT, a combination of a statin and ezetimibe reduces LDL-C levels and the risk of MACE equally in women and men [158]. In the cardiovascular endpoint trial IMPROVE-IT, ezetimibe decreased MACE rates similarly in women and men [159]. Although a meta-analysis revealed significantly greater changes in LDL-C (*p* = 0.0066), non-HDL-C, TC, TG (*p* < 0.0001), and apoB levels (*p* = 0.0055) in men compared to women on statin and ezetimibe, the differences were only ≤2% [160]. Interestingly, a comparison of high-intensity statin monotherapy with a combination of moderate-intensity statin and ezetimibe found both treatment strategies equally efficient in reducing cardiovascular event rates regardless of sex. Of note, discontinuation or dose reduction rates were lower in the latter compared to the former group in both women and men [161]. Moreover, among patients with ASCVD or HeFH treated with statins and bempedoic acid, greater placebo-corrected reductions in LDL-C (21.2 vs. 17.4%, *p* = 0.044), non-HDL-C (17.3 vs. 12.1%, *p* = 0.003), TC (13.8 vs. 10.5%, *p* = 0.012), and apoB levels (16 vs. 11.3%, *p* = 0.004) were observed in women than men. Conversely, comparable results were reported among both sexes in patients receiving bempedoic acid along with low-dose or no statin [162]. Most importantly though, bempedoic acid reduced LDL-C levels and cardiovascular risk similarly in women and men in the cardiovascular endpoint trial Clear Outcomes [163].

Sex differences are most pronounced in LDL-C reduction with PCSK9 inhibitor treatment. A greater reduction in LDL-C levels was reported in men than in women on evolocumab at 4 weeks (58% vs. 52%, *p* < 0.001) [164]. In addition, a recent observational, prospective study evaluated the sex-specific response to evolocumab in a real-world setting and found higher LDL-C reductions in men during 30-month follow-up. Interestingly, Lp(a) levels decreased similarly in both women and men [165]. Nevertheless, relative risk reductions in the primary endpoint and key secondary endpoint in the evolocumab cardiovascular endpoint trial Fourier were similar for both sexes [164]. Correspondingly, alirocumab treatment was associated with a higher LDL-C decline in men than women in placebo-controlled (60 vs. 48.3%) and ezetimibe-controlled trials (50.9 vs. 42.3%). Although only 36.5% of women compared to 58.7% of men achieved on-treatment LDL-C levels <50 mg/dL, no sex differences occurred in the risk of MACE in the alirocumab cardiovascular endpoint trial Odyssey Outcomes [166]. These results were confirmed in meta-analyses evaluating the efficacy of PCSK9 inhibitors in women [167,168], as well as in real-world registries [169,170]. The latter reported lower LDL-C reduction in women than men both among patients with (48% vs. 61%, *p* < 0.001) and without HeFH (55% vs. 62%, *p* = 0.015) [169]. Of note, despite differences in circulating PCSK9 levels, no differences in LDL-C reduction were observed between pre- and post-menopausal women on PCSK9 inhibitors [169,170].

#### 4.2.2. Real-World Lipid-Lowering Therapy in Women

Given the comparable efficacy of lipid-lowering agents in women and men, current guidelines do not recommend sex-specific treatment regimens [3,4,5] and hence should be followed equally regardless of sex. However, real-world data demonstrate that women are less likely than men to receive any statin (OR: 0.70, *p* < 0.001). Even if treated, women receive lower than the guideline-recommended intensity statin more often than men (OR: 0.82, *p* < 0.001) [9]. In addition, women with CAD treated with statins are less often evaluated by cardiologists (62 vs. 67.4%, *p* < 0.001), whereas such an evaluation is associated with higher statin use (OR: 2.535, *p* < 0.001) [171]. Consequently, women achieve the guideline-recommended LDL-C levels less often than men. For instance, in a recent study from Portugal, women were found to be 22% less likely to reach the LDL-C goal than men [19]. Higher LDL-C levels were found in women both in primary and secondary prevention settings (age-adjusted difference: 0.3 and 0.28 mmol/L, respectively) [20]. However, it should be pointed out that cardiovascular risk-based LDL-C goal attainment is generally low, in both men and women, varying between 20 and 30%, as consistently reported across Europe [172,173,174] and in the United States [175].

Considering patients with FH, a German registry showed that men with FH are more likely to receive high-intensity statin treatment (OR: 1.52, *p* < 0.05) [12]. Correspondingly, women with FH less often reach LDL-C levels <100 mg/dL (20 vs. 29%, *p* < 0.001), consistently among FH patients with ASCVD, without ASCVD, and those on statin treatment (ORs: 0.70, 0.68, and 0.74, respectively) [21]. The most prominent sex differences have been shown in patients with HeFH, with women being on LLT less often than men (58.4 vs. 61.1%, *p* < 0.001). If treated, women with HeFH take the highest statin dose (16.6 vs. 13.1%, *p* < 0.001), ezetimibe (23.4 vs. 25.9%, *p* < 0.0001), and PCSK9 inhibitors (2.5 vs. 3.5%, *p* = 0.0002) less frequently than men [11]. Consequently, women with HeFH have higher on-treatment LDL-C levels (125 vs. 116 mg/dL, *p* = 0.02), especially among those with premature ASCVD (135 vs. 109 mg/dL, *p* = 0.005) [176]. Importantly, a significantly stronger association between higher pre-treatment LDL-C levels and not reaching the LDL-C goal was found in women compared to men with HeFH, both among patients with (OR: 0.35 vs. 0.64, *p* = 0.02) and without ASCVD (OR: 0.70 vs. 0.83, *p* = 0.013) [92].

Alarmingly, women are approximately 2.5 years older when diagnosed with HeFH [11] and are commenced on LLT later in life than men (42.3 vs. 37.5 years of age, *p* < 0.0001) [177]. In addition, women with FH experience a median of 2.3-year-long pregnancy-related off-statin periods, contributing to a greater total LDL-C burden [178]. Of note, maternal hypercholesterolemia during pregnancy is associated with higher LDL-C levels in offspring (MD: 0.4 mmol/L, *p* < 0.01) and hence might not only increase the cardiovascular risk of the mother but also of the child [179]. Overall, women with HeFH suffer from greater excess cardiovascular morbidity compared to men with HeFH (standardized morbidity ratio: 7.55 vs. 6.83), especially among patients aged 30–50 years (15.04 vs. 10.03) [177]. In contrast, a recent analysis of the largest global dataset of patients with HoFH reported a comparable age at diagnosis in both, women and men (median: 13 vs. 11 years for women and men, respectively). Furthermore, no sex differences occur in LLT intensity and LDL-C goal attainment. Of note, the latter is generally low, with only 4.0% of women and 3.1% of men achieving their LDL-C goal. HoFH is associated with extremely elevated LDL-C levels, often accompanied by skin and tendon xanthomas and thus is diagnosed in childhood or adolescence and requires intensive treatment at specialized lipid centers. This may partially explain the lack of sex differences, which have been consistently reported in patients with HeFH [93].

Regarding other aspects of LLT, women discontinue statin treatment more often than men (10.9 vs. 6.1%, *p* < 0.001) [9]. Side effects are the most frequent reason for stopping or switching to another statin in both sexes, but women are more likely to report them. A higher rate of new or worsening muscle symptoms as side effects of statin treatment were found in women than men (31 vs. 26%, *p* < 0.01) [22]. Importantly, women with a history of statin use are less likely than men to try another statin [9]. Furthermore, sex differences occur in adherence to statin treatment [23], with women being less likely to take statins as prescribed (67 vs. 71%, *p* = 0.007) but more likely to skip a dose or not to fill a prescription (35 vs. 31%, *p* = 0.02).

Considering the patient–physician relationship, women are less likely to be informed about the risk associated with elevated LDL-C levels, which might contribute to a greater proportion of women dissatisfied with their treatment [22] and questioning statin efficacy and safety [9]. Correspondingly, lower adherence to statin treatment was also reported in women compared to men with FH [24]. Moreover, women with FH receive adequate information about FH (68.9 vs. 80.2%, *p* = 0.011) and the associated risks (71.1 vs. 80.7%, *p* = 0.033) less frequently than men, as well as less often agree that statins are safe (44.8 vs. 60%, *p* = 0.003). At the same time, women with FH are more concerned about their high LDL-C levels than men (67.5 vs. 56.5%, *p* = 0.024) [25].

### 4.3. Key Messages

Altogether, the prevalence of cardiovascular risk factors differs in women and men. Women might have a greater burden related to LDL-C, Lp(a), and TG levels, especially among patients with HeFH. Women are exposed not only to traditional cardiovascular risk factors but also those associated with reproductive health and psychosocial status. Among patients with ASCVD, women and men exhibit different plaque morphology and pathophysiology. Moreover, unusual disease manifestations in women can negatively influence the diagnostic process, treatment course, and clinical outcome. Therefore, global female cardiovascular risk might be underestimated, leading to delayed LLT initiation, as a part of ASCVD prevention.

No sex differences occur in the risk of MACE following LLT and hence both women and men benefit equally from LLT. Despite comparable LLT efficacy, women are less likely than men to be offered LLT, including high-intensity statins, ezetimibe, and PCSK9 inhibitors. Consequently, women have higher on-treatment LDL-C levels and achieve their risk-based LDL-C goal less often than men. A similar trend is observed among patients with HeFH. In addition, women with HeFH are diagnosed and started on LLT later in life and are exposed to pregnancy-associated off-treatment periods, which contribute to greater LDL-C burden and cardiovascular morbidity compared to men. Although women with HoFH are treated similarly to men, they face extremely high cardiovascular risk and rarely achieve the LDL-C goal. Moreover, women are less adherent to statin treatment, mostly due to side effects, and have less satisfying patient–physician relationships than men.

## 5. Conclusions and Future Directions

**Figure 5:**
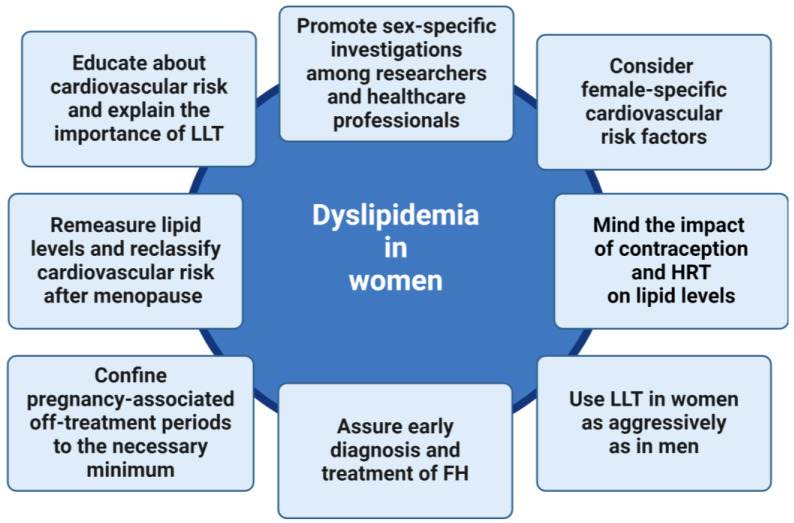
Key messages about the management of dyslipidemia in women. FH—familial hypercholesterolemia; HRT—hormone replacement therapy; LLT—lipid-lowering therapy. Created with BioRender.com.

The key messages about the management of dyslipidemia in women are summarized in Figure 5.

Men still constitute the majority of clinical trial participants, even in recent years. For instance, trials investigating novel LDL-C-lowering inclisiran and Lp(a)-lowering olpasiran include approximately 32% women [180,181]. Consequently, most of the available data on MACE reduction with LLT is derived from men. Female underrepresentation may partly result from a lower willingness of women to participate in trials due to greater concerns about possible harm or transportation issues. Interestingly though, more women are included in trials with female corresponding authors, indicating the important role the sex of researchers may play in resolving this conundrum [182]. To gain a better understanding of sex differences in dyslipidemia and LLT efficacy and safety, greater representation of women in clinical trials, as well as sex-specific research, must be endorsed. Further studies are necessary to investigate, among others, (i) the role of sex-specific hormonal and genetic determinants in dyslipidemia and ASCVD, (ii) the relationship between menstrual cycle and lipid profile, (iii) the meaning of menopausal transition for ASCVD prevention, (iv) the burden and treatment of dyslipidemia in pregnant women, (v) the impact of contraception and HRT on dyslipidemia and cardiovascular risk, (vi) the influence of Lp(a) and TG levels on cardiovascular risk in women, and (vii) sex disparities in the response to novel lipid-lowering agents.

Based on the presented data, women with dyslipidemia could benefit from sex-specific recommendations. For instance, lipoprotein-level monitoring, especially regarding TG levels, seems reasonable in women taking contraception or HRT. Given the menopause-related changes, repeated measurement of lipoprotein levels, including Lp(a), could lead to reclassification of the female cardiovascular risk after menopausal transition. Correspondingly, considering additional cardiovascular risk factors, such as those related to reproductive health or psychosocial status, along with traditional ones, can contribute to more precise cardiovascular risk stratification in women. Among patients on LLT, the use of combination treatment might prevent side effects of statin treatment and hence improve female adherence. In addition, due to pregnancy-associated off-treatment periods, early LLT initiation and strict follow-up to minimize time off-treatment seem rational, especially in women with FH.

Furthermore, to ensure that both women and men receive equitable LLT, there is a need to promote sex-specific research outcomes among healthcare professionals. Specifically, physicians treating patients with dyslipidemia should be educated on sex differences in lipid and cardiovascular risk profiles and disease manifestations. More attention should also be brought to the impact of sex and gender on treatment course and adherence. Hereby, physicians must be encouraged not to delay LLT initiation in women and to use the guideline-recommended LLT intensity both in women and men. Treatment equality, along with a need for earlier diagnosis in women, must be advocated especially considering patients with HeFH. Finally, to raise female awareness of the cardiovascular risk associated with dyslipidemia, appropriate actions must be undertaken both in clinical practice and at the institutional level. Spreading the knowledge about LLT’s importance, efficacy, and safety is essential to reduce the LDL-C burden in women. It is long overdue that national and international guidelines address the issue of sex differences in ASCVD prevention and tailor their recommendation accordingly [17,83,152].

## Data Availability

Not applicable.
